# Components of the tumor immune microenvironment based on m‐IHC correlate with prognosis and subtype of triple‐negative breast cancer

**DOI:** 10.1002/cam4.6718

**Published:** 2023-12-07

**Authors:** Luyi Lin, Haiming Li, Xin Wang, Zezhou Wang, Guanhua Su, Jiayin Zhou, Shiyun Sun, Xiaowen Ma, Yan Chen, Chao You, Yajia Gu

**Affiliations:** ^1^ Department of Radiology Fudan University Shanghai Cancer Center Shanghai China; ^2^ Department of Pathology Fudan University Shanghai Cancer Center Shanghai China; ^3^ Department of Cancer Prevention Fudan University Shanghai Cancer Center Shanghai China; ^4^ Department of Oncology Shanghai Medical College, Fudan University Shanghai China; ^5^ Shanghai Municipal Hospital Oncological Specialist Alliance Shanghai China; ^6^ Department of Breast Surgery, Key Laboratory of Breast Cancer in Shanghai Fudan University Shanghai Cancer Center Shanghai China; ^7^ Division of Cancer and Stem Cell School of Medicine at University of Nottingham Nottingham UK

**Keywords:** multiplex IHC, prognosis, subtype, triple‐negative breast cancer, tumor immune microenvironment

## Abstract

**Background and Aim:**

The spatial distribution and interactions of cells in the tumor immune microenvironment (TIME) might be related to the different responses of triple‐negative breast cancer (TNBC) to immunomodulators. The potential of multiplex IHC (m‐IHC) in evaluating the TIME has been reported, but the efficacy is insufficient. We aimed to research whether m‐IHC results could be used to reflect the TIME, and thus to predict prognosis and complement the TNBC subtyping system.

**Methods:**

The clinical, imaging, and prognosis data for 86 TNBC patients were retrospectively reviewed. CD3, CD4, CD8, Foxp3, PD‐L1, and Pan‐CK markers were stained by m‐IHC. Particular cell spatial distributions and interactions in the TIME were evaluated with the HALO multispectral analysis platform. Then, we calculated the prognostic value of components of the TIME and their correlations with TNBC transcriptomic subtypes and MRI radiomic features reflecting TNBC subtypes.

**Results:**

The components of the TIME score were established by m‐IHC and demonstrated positive prognostic value for TNBC (*p* = 0.0047, 0.039, <0.0001 for DMFS, RFS, and OS). The score was calculated from several indicators, including Treg% in the tumor core (TC) or stromal area (SA), PD‐L1^+^ cell% in the SA, CD3 + cell% in the TC, and PD‐L1^+^/CD8^+^ cells in the invasive margin and SA. According to the TNBC subtyping system, a few TIME indicators were significantly different in different subtypes and significantly correlated with MRI radiomic features reflecting TNBC subtypes.

**Conclusion:**

We demonstrated that the m‐IHC‐based quantitative score and indicators related to the spatial distribution and interactions of cells in the TIME can aid in the accurate diagnosis of TNBC in terms of prognosis and classification.

## 1 INTRODUCTION

Triple‐negative breast cancer (TNBC) without both hormone receptor and HER2 expression is considered a poor prognostic subtype because of the lack of appropriate therapeutic targets.^1^ Currently, immunomodulators, such as those targeting the PD‐1/PD‐L1 pathway, in addition to first‐line chemotherapy play an effective role in the treatment of some TNBC patients, but these therapies have poor efficacy in other patients due to the complexity of the tumor immune microenvironment (TIME) in TNBC.^2^, ^3^, ^4^, ^5^ In TNBC lesions, PD‐L1 is predominantly located on tumor‐infiltrating immune cells and typically inhibits antitumour immune responses, so the spatial distribution and interactions of cells in the TIME influence the efficacy of immunomodulators.^6^, ^7^, ^8^

The multiplex immunohistochemistry (m‐IHC) method is an effective technique for evaluating the tumor TIME by identifying the spatial distribution and interactions of specific cells that significantly influence the clinical outcome.^9^ In the field of breast cancer, a few studies have used m‐IHC to detect differences in components of the TIME between different molecular types, the relationship between components of the TIME and the efficacy of neoadjuvant therapy, and the relationship between PD‐1^+^ CD8^+^ double‐positive cells and prognosis.^10^, ^11^, ^12^ However, due to the extremely complex cellular composition of the TIME, the impact of the spatial relationships and interactions of specific cells, such as Tregs, CD8^+^ cells, and PD‐L1^+^ cells, on the prognosis of TNBC remains unclear.

Recent studies have found that TNBC can be further divided into four subtypes with different prognoses and potential treatment targets, namely, luminal androgen receptor (TNBC‐LAR), immunomodulatory (TNBC‐IM), basal‐like immune‐suppressed (TNBC‐BLIS), and mesenchymal‐like (TNBC‐MES), according to transcriptome levels.^13^ Further studies have found differences in AR, CD8, FOXC1, and DCLK expression among the four TNBC subtypes,^14^ and a noninvasive radiomic model has been established to distinguish the different TNBC subtypes.^15^ In addition, clinical trials have demonstrated that immunomodulators are effective in the treatment of some TNBC subtypes,^16^, ^17^ which is helpful for personalized treatment.

In this study, we investigated the spatial distribution and interactions of specific cells in the TIME in situ based on the m‐IHC method to identify appropriate indicators of the clinical prognosis of TNBC. We explored the relationships of several TIME indicators with the TNBC transcriptome subtypes as well as MRI radiomic features that could reflect the subtypes. Our aims were to consider the TNBC subtyping system from a micro to a macro perspective. These achievements will provide more precise tools to better predict and personalize TNBC treatment.

## 2 METHODS

### 2.1 Patients and study design

This study was a retrospective observational study of a consecutive case series from a single institution and was conducted in accordance with the Declaration of Helsinki (revised in 2013). All analyses were approved by the independent ethics committee/institutional review board of Fudan University Shanghai Cancer Center (050432‐4‐1911D), and written informed consent was obtained from each patient. This study included TNBC patients who were treated at Fudan University Cancer Center in Shanghai from August 1, 2009 to May 31, 2015, and met the following criteria for inclusion: (1) no evidence of distant metastasis at the time of diagnosis, (2) complete breast‐enhanced MRI performed before biopsy, and clinicopathologic data were available, (3) postoperative tumor specimens were collected at our hospital. The clinicopathologic features included age, menopausal status, tumor size, lymph node metastasis status, and Ki67 index. During this period, a total of 202 TNBC patients were collected, and postoperative specimens from part of the patients were stained with m‐IHC. After discarding samples that did not pass quality check, a final of 86 cases were available for further analysis.

All patients were followed up after surgery and adjuvant radiotherapy or chemotherapy at our hospital. Follow‐up within this cohort of patients was completed on June 30, 2017, and the median length of follow‐up was 45.8 months (interquartile range, 34.4–59.8 months). Clinical prognostic information, including survival status and metastasis, was summarized, and overall survival (OS), relapse‐free survival (RFS), and distant metastasis‐free survival (DMFS) were calculated. OS was defined as the time from diagnosis to death from any cause. RFS was defined as the time from diagnosis to first recurrence or a diagnosis of contralateral breast cancer. DMFS was defined as the time from diagnosis to distant metastasis (such as liver and lung metastasis). Patients without events were censored from the time point of the last follow‐up. OS was selected as the primary outcome, and RFS and DMFS were selected as secondary outcomes.

These TNBC cases were classified into the four subtypes, namely, TNBC‐LAR, TNBC‐IM, TNBC‐BLIS, and TNBC‐MES, by transcriptomic analysis in our previous study. MRI radiomic features of these TNBC lesions were validated to reflect the four transcriptomic subtypes in another previous study.^15^

**FIGURE 1:**
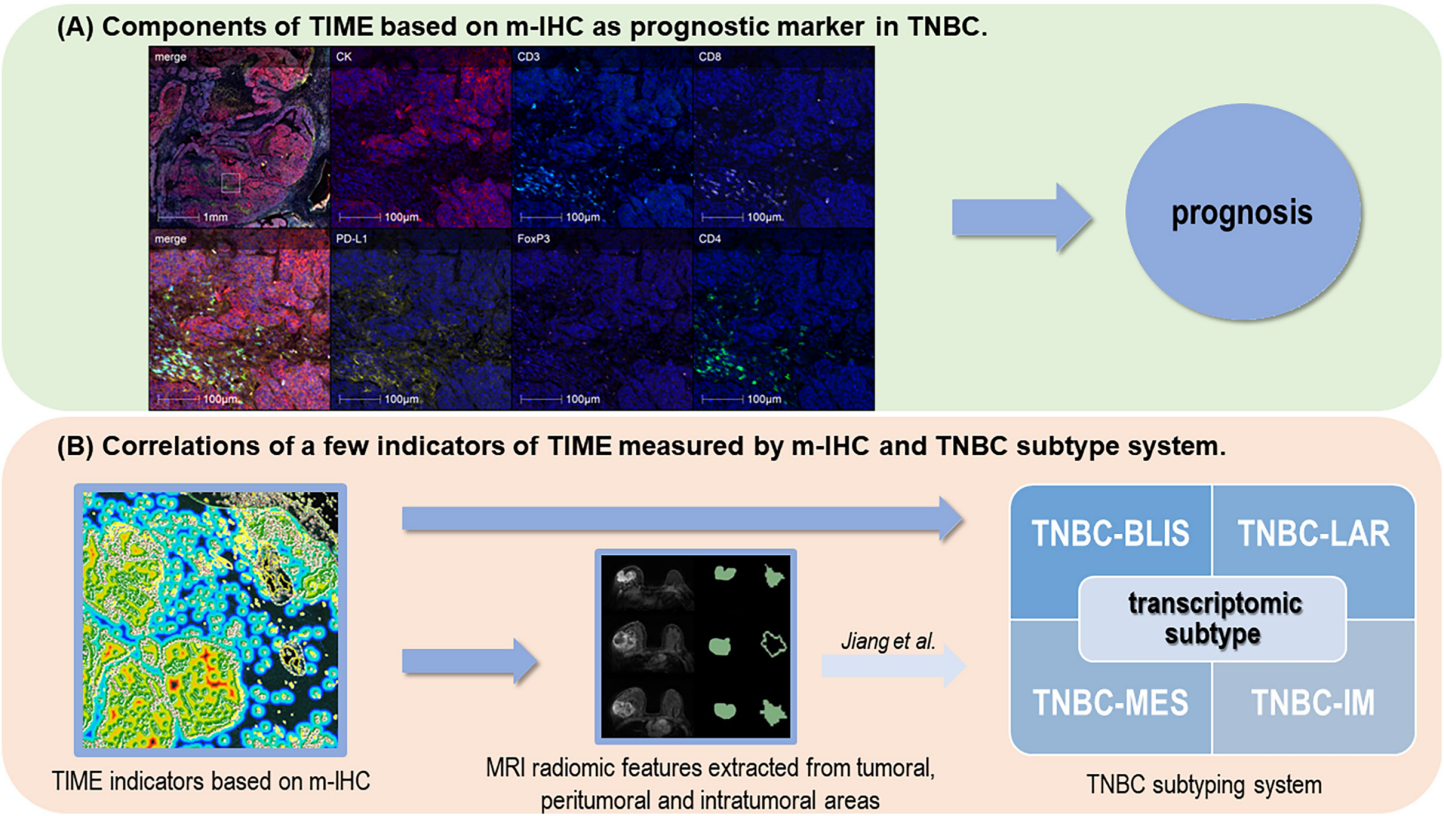
Overview of this research. (A) Components of the tumor immune microenvironment (TIME) based on m‐IHC were found to be effective predictive markers of prognosis in TNBC patients. Markers of m‐IHC were Pan‐CK, CD8, PDL1, CD3, CD4, and FoxP3. Prognosis included overall survival (OS), relapse‐free survival (RFS), and distant metastasis‐free survival (DMFS). (B) A few indicators of TIME measured by m‐IHC could complement the TNBC subtyping system. The TIME indicators correlated with TNBC transcriptomic subtypes, namely, the TNBC‐luminal androgen receptor (TNBC‐LAR), TNBC‐immunomodulatory (TNBC‐IM), TNBC‐basal‐like immune‐suppressed (TNBC‐BLIS), and TNBC‐mesenchymal‐like (TNBC‐MES) subtypes. The TIME indicators also correlated with MRI radiomic features reflecting TNBC subtypes.^15^

The study design is shown in Figure 1. We first explored the prognostic value of the TIME indicators and scores measured by m‐IHC in TNBC patients. Then, we explored the relationships between a few important TIME indicators with the TNBC transcriptome subtype and MRI radiomic features reflecting TNBC subtypes. Details of the MRI radiomic features are shown in the supplementary methods and Table S2.

### 2.2 M‐IHC details

Paraffin sections of TNBC cases obtained from postsurgical specimens were subjected to sequential rounds of primary antibody staining. Based on previous research, six markers, CD3, CD4, CD8, FoxP3, PD‐L1, and Pan‐CK, were selected. The multiplex panel of primary antibodies included rabbit monoclonal [SP239] antibodies to CD8 alpha (ab178089, Abcam, 1:100), rabbit monoclonal [EPR6855] antibodies to CD4 (ab133616, Abcam, 1:400), rabbit monoclonal [CAL10] antibodies to PD‐L1 (ab237727, Abcam, 1:250), mouse monoclonal [C‐11] antibodies to pancytokeratin (ab7753, Abcam, 1:150), rabbit monoclonal [SP7] antibodies to CD3 (ab16669, Abcam, 1:150), rabbit monoclonal [EPR22102‐37] antibodies to FoxP3 (ab215206, Abcam, 1:150), and DAPI. Secondary antibody incubation was carried out with a mix of horseradish peroxidase‐conjugated anti‐mouse or anti‐rabbit secondary antibodies (Akoya Bioscience), and tyramide signal amplification visualization was performed with the fluorophores Opal 520, 620, 570, 690, 480, and 780 (Akoya Bioscience).

### 2.3 Components of the TIME measured with the HALO platform

**FIGURE 2:**
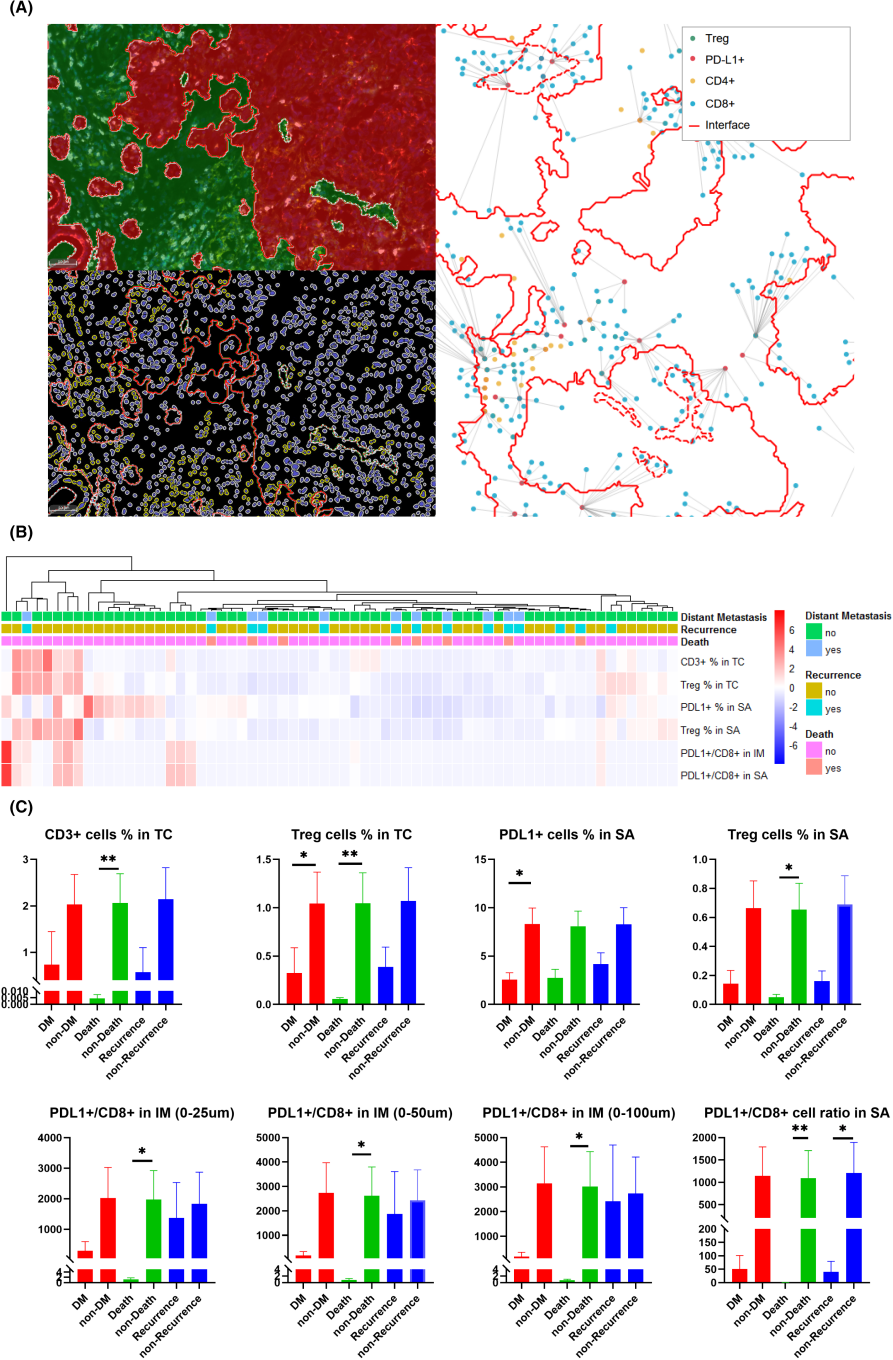
(A) Measurement of the components of the TIME indicators based on the HALO multispectral analysis platform. Classification of the TC (green) and SA (red) and the phenotypes of particular cells based on the HALO platform are shown; CD8^+^ cells, for example, are shown in yellow. Spatial analysis of the components of the TIME based on the HALO platform is also shown. (B) Cluster heatmap of the positive cell fraction of the marker cells for these TNBC patients and their clinical outcome. (C) Univariate analysis of components of TIME indicators between DM, death, recurrence and opposite groups. * means *p* < 0.05, ** means *p* < 0.01. DM, distant metastasis; IM, invasive margin; SA, stromal area; TC, tumor core; TIME, tumor immune microenvironment; TNBS, triple‐negative breast cancer.

Multiplexed immunofluorescence slides were scanned on a Vectra‐Polaris Automated Quantitative Pathology Imaging System (Akoya Biosciences). The phenotype and spatial measurement of a particular cell type were determined with HALO software. As shown in Figure 1A, an appropriate threshold was set to perform spectral unmixing. Cells were segmented based on nuclear detection and were automatically subclassified as CD3^+^, CD4^+^, CD8^+^, PD‐L1^+^, Pan‐CK^+^, and Treg (CD4^+^ FoxP3^+^) cells by a random tree algorithm classifier. The tumor core (TC) and stromal area (SA) were automatically segmented based on Pan‐CK^+^ cells with the HALO platform, as shown in Figure 2A. Moreover, segmentation of the TC and SA was verified by pathological experts based on traditional HE staining. Spatial measurements included four categories: the percentage of particular cells in the TC or SA, the particular cell density in the invasion margin (IM), the ratio of particular cells in the IM, and normalized particular cells around CD8^+^ cells in the SA (Figure 2A; Table S1). With reference to previous m‐IHC‐based TIME‐related studies in other tumors, the IM was defined as ring shapes of 25, 50, and 100 μm outside the margin between the TC and SA.^18^, ^19^

### 2.4 Statistical analysis

The data were analyzed using SPSS 25.0 software and R studio 4.1.2. None of the indexes of the TIME components conformed to a normal distribution, according to the Kolmogorov–Smirnova test. The Mann–Whitney *U*‐test was used for univariate analysis, and Kaplan–Meier curves were used for survival analysis. A Cox proportional hazards model was used for multivariate analysis. The association between components of the TIME and radiomic features was evaluated by the Spearman correlation coefficient. All tests were two‐sided, and *p* < 0.05 was considered statistically significant.

## 3 RESULTS

### 3.1 Overview of clinical characteristics and prognosis

The clinical characteristics of the 86 TNBC patients are shown in Table 1. The average age of the patients was 53.12 ± 10.12 years. Breast conservation surgery was performed in 1 patient, and radical surgery was performed in 85 patients. All patients received adjuvant chemotherapy after surgery; 25 patients received adjuvant radiotherapy, and 61 patients did not. In terms of clinical outcomes, 9 patients died (10.5%), 16 patients relapsed (18.6%), and 12 patients (14.0%) had distant metastasis (DM). Regarding clinical features, only the N stage was significantly different (*p* < 0.001) between the DM group and the non‐DM group.

**TABLE 1:** Clinical characteristics at diagnosis of all the TNBC cases and their prognosis.

Age	Mean ± SD/*N*	Rate
	53.12 ± 10.12	
Menopause		
Pre	22	25.6%
Post	64	74.4%
*T*		
1	36	41.9%
2	47	54.7%
3	3	3.5%
*N*		
0	53	61.6%
1	19	22.1%
2	10	11.6%
3	4	4.7%
Ki67%		
<20	3	3.5%
≥20	83	96.5%
Adjuvant radiation therapy		
No	61	70.9%
Yes	25	29.1%
Prognosis		
Death	9	10.5%
M/R	16	18.6%
DM	12	14.0%

Abbreviations: DM, distance metastasis; M/R, metastasis/recurrence; TNBS, triple‐negative breast cancer.

### 3.2 Components of the TIME effectively predict the prognosis of TNBC patients

**FIGURE 3:**
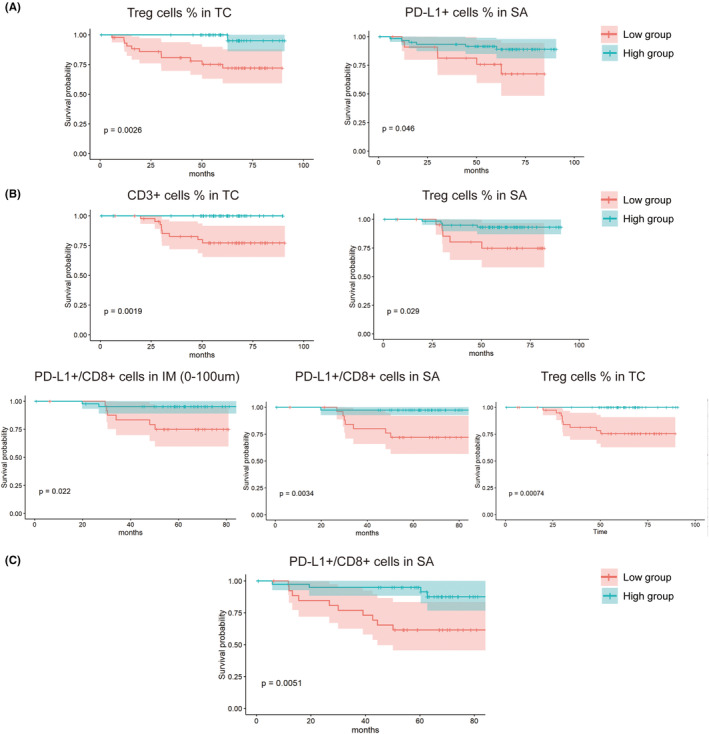
Survival analysis of TIME indicators measured by m‐IHC and clinical outcome of DMFS (A), OS (B), and RFS (C). DMFS, distant metastasis‐free survival; OS, overall survival; RFS, relapse‐free survival; TIME, tumor immune microenvironment.

The spatial distribution and interaction of important cells of the TIME evaluated by the m‐IHC method were compared between the DM, death, or recurrence group and the opposite group (Table 2). The percentage of marker‐positive cells among all cells was quantitatively analyzed and illustrated in a heatmap. With reference to the expression of particular cells in tumors, we clustered the patients and examined their prognosis (Figure 2B). Univariate analysis showed a significantly higher Treg% in the TC (1.04 ± 0.33 vs. 0.32 ± 0.26, *p* = 0.018; 1.05 ± 0.32 vs. 0.06 ± 0.01, *p* = 0.004) in the non‐DM and non‐death groups and a significantly higher PD‐L1^+^/CD8^+^ cell ratio in the SA (1095.88 ± 614.88 vs. 0.37 ± 0.12, *p* = 0.005; 1209.44 ± 683.31 vs. 40.00 ± 39.41, *p* = 0.0420) in the non‐death and non‐recurrence groups. In addition, a higher PD‐L1^+^ cell% in the SA (8.34 ± 1.63 vs. 2.56 ± 0.72, *p* = 0.033), CD3^+^ cell% in the TC (2.07 ± 0.62 vs. 0.005 ± 0.00, *p* = 0.004), Treg% in the SA (0.66 ± 0.18 vs. 0.05 ± 0.01, *p* = 0.047), and PD‐L1^+^/CD8^+^ cell ratio in the IM (0–25, 0–50, and 0–100 μm: 1974.30 ± 948.31 vs. 1.17 ± 0.65, *p* = 0.028; 2621.38 ± 1175.48 vs. 0.86 ± 0.39, *p* = 0.025; and 3021.97 ± 1410.13 vs. 0.73 ± 0.28, *p* = 0.025) were found in the non‐DM, non‐death, or non‐recurrence groups compared with the other groups (Figure 2C). The results of multivariate COX regression analysis of clinicopathological and components of TIME features were shown in Table 3. In addition, the optimal cutoff values of the TIME indicators were found based on the Youden index of the ROC curve. Survival analysis confirmed significantly different prognoses in patients with different TIME performances. Compared to low values of the TIME indicators, a high Treg% in the TC, PD‐L1^+^/CD8^+^ cell ratio in SA, PD‐L1^+^ cell% in SA, CD3^+^ cell% in TC, Treg% in SA, and PD‐L1^+^/CD8^+^ cell ratio in the IM group were associated with significantly higher DMFS, OS, or RFS (all *p* < 0.05), as shown in Figure 3A–C.

**TABLE 2:** Differences of components of TIME between DM and non‐DM, non‐death and death, non‐relapse and relapse in TNBC cases.

	DM		*p*	Death		*p*	Recurrence		*p*
	Non‐DM	DM		Non‐death	death		Non‐*R*	*R*	
	Mean ± SED	Mean ± SED		Mean ± SED	Mean ± SED		Mean ± SED	Mean ± SED	
CD3^+^ cells % in TC	2.03 ± 0.65	0.73 ± 0.71	0.104	2.07 ± 0.62	0.005 ± 0.003	0.004*	2.14 ± 0.68	0.57 ± 0.53	0.084
Treg cells % in TC	1.04 ± 0.33	0.32 ± 0.26	0.018*	1.05 ± 0.32	0.06 ± 0.01	0.004*	1.07 ± 0.34	0.39 ± 0.21	0.149
PDL1^+^ cells % in SA	8.34 ± 1.63	2.56 ± 0.72	0.033*	8.09 ± 1.57	2.75 ± 0.88	0.103	8.30 ± 1.72	4.17 ± 1.17	0.226
Treg^+^ cells % in SA	0.66 ± 0.19	0.14 ± 0.09	0.122	0.66 ± 0.18	0.05 ± 0.01	0.047*	0.69 ± 0.19	0.16 ± 0.06	0.484
PDL1^+^/CD8^+^ in IM (0–25 μm)	2024.20 ± 1000.77	298.91 ± 297.45	0.637	1974.30 ± 948.31	1.17 ± 0.65	0.028*	1836.55 ± 1033.69	1371.98 ± 1157.65	0.461
PDL1^+^/CD8^+^ in IM (0–50 μm)	2731.57 ± 1238.14	164.61 ± 163.49	0.444	2621.38 ± 1175.48	0.86 ± 0.39	0.024*	2431.65 ± 1248.61	1868.87 ± 1743.55	0.295
PDL1^+^/CD8^+^ in IM (0–100 μm)	3147.42 ± 1482.86	174.62 ± 173.64	0.429	3021.97 ± 1410.13	0.73 ± 0.28	0.025*	2737.00 ± 1476.36	2417.54 ± 2283.78	0.290
PDL1^+^/CD8^+^ cell ratio in SA	1144.66 ± 647.42	50.84 ± 50.16	0.279	1095.88 ± 614.88	0.37 ± 0.12	0.005*	1209.44 ± 683.31	40.00 ± 39.41	0.042*

Abbreviations: DM, distant metastasis; IM, invasive margin; SA, stromal area; TC, tumor core; TNBC, triple negative breast cancer; TIME, tumor immune microenvironment. * means *p* < 0.05.

**TABLE 3:** Multivariate analysis of clinicopathology, components of TIME features and prognosis, including DM, death and recurrence in TNBC patients.

	DM			Death			Recurrence		
	*p*	HR	95% CI	*p*	HR	95% CI	*p*	HR	95% CI
*N* stage	0.016*	1.858	1.124–3.071	/	/	/	0.063	1.530	0.977–2.397
Treg cells % in TC	0.041*	0.115	0.015–0.911	/	/	/	/	/	/
PDL1^+^/CD8^+^ cell ratio in SA	/	/	/	0.023*	0.087	0.011–0.711	0.029*	0.266	0.081–0.872

Abbreviations: DM, distant metastasis; SA, stromal area; TC, tumor core; TNBC, triple negative breast cancer; TIME, tumor immune microenvironment. * means *p* < 0.05.

### 3.3 TIME score predicts the prognosis of TNBC patients

**FIGURE 4:**
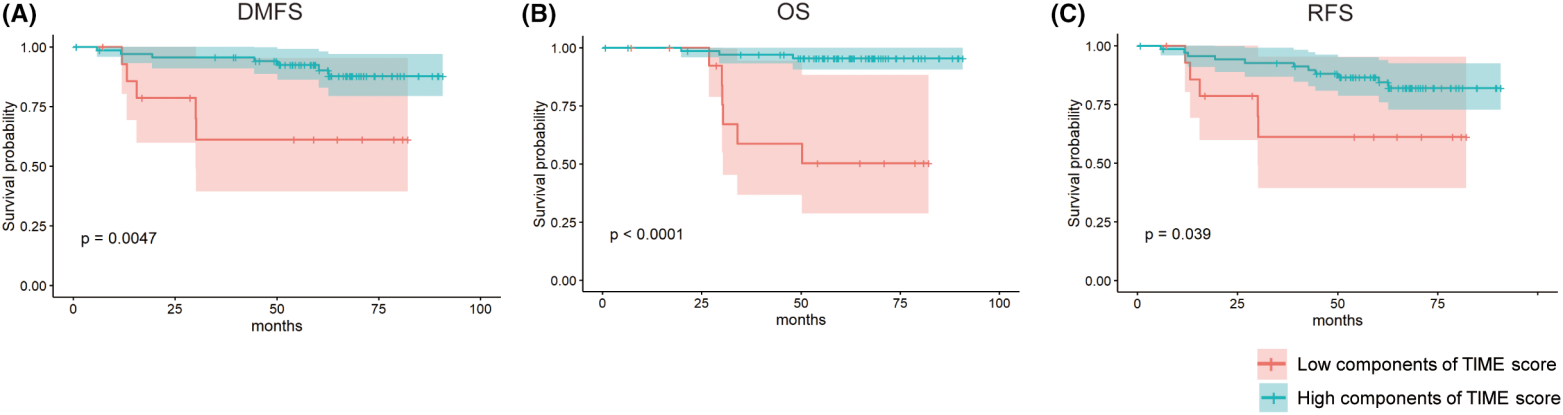
Survival analysis of the TIME score and clinical outcome, including (A) DMFS, (B) OS, and (C) RFS in TNBC patients. DMFS, distant metastasis‐free survival; OS, overall survival; RFS, relapse‐free survival; TIME, tumor immune microenvironment; TNBS, triple‐negative breast cancer.

The TIME score was established according to the significantly different indicators of the TIME assessed by the above univariate analysis. For the index of IM of different ranges, we selected the index with the largest difference between groups, that is, PDL1^+^/CD8^+^ in IM (0–50 μm), as the factor to calculate the TIME component score. Considering the clinical outcomes of DMFS, OS, and RFS, low or high Treg cell % in the TC, PD‐L1^+^ cell % in the SA, CD3^+^ cell % in the TC, Treg cell % in the SA, PD‐L1^+^/CD8^+^ cells in the IM (0–50 μm), and PD‐L1^+^/CD8^+^ cells in the SA were divided by the best cutoff determined according to the Youden Index. Each of the six indicators had a weight of one point. Finally, each TNBC patient received points based on the TIME components for a total TIME score of 0–6 points. Patients with a score of 0–1 were regarded as having a low TIME score, while others were regarded as having a high TIME score for further analysis. Survival analysis showed that TNBC patients in the low TIME score group had a significantly poorer prognosis than those in the high TIME score group in terms of DMFS (*p* = 0.0047), OS (*p* < 0.0001), and RFS (*p* = 0.039) (Figure 4).

#### 3.3.1 Components of the TIME were correlated with the molecular subtype of TNBC

**FIGURE 5:**
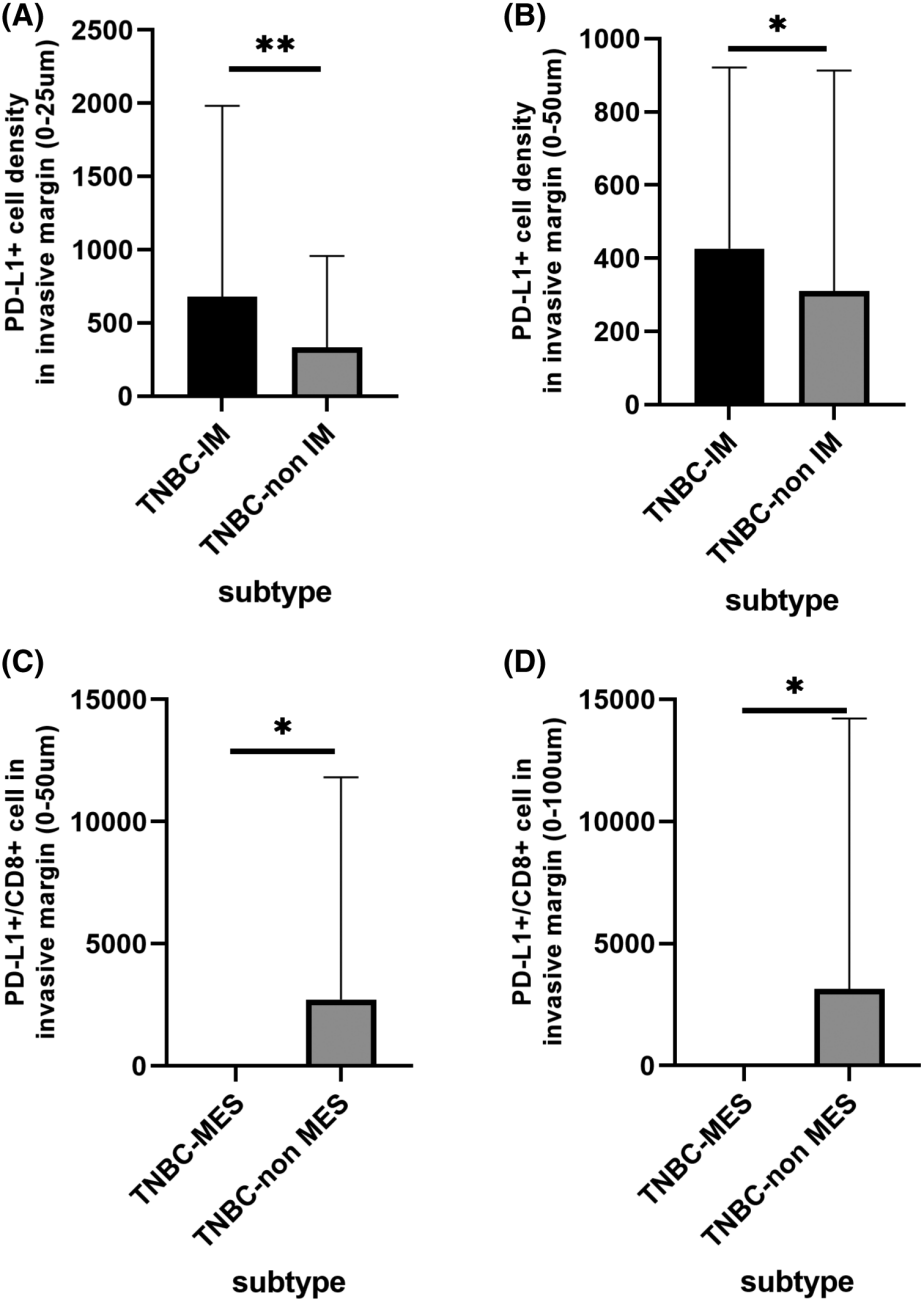
Several indicators of the TIME were correlated with the molecular subtype of TNBC patients. (A, B) The PD‐L1^+^ cell density in the IM significantly differed between TNBC‐IM and TNBC‐non IM patients. (C, D) PD‐L1^+^/CD8^+^ cells in the IM were significantly different between TNBC‐MES and TNBC‐non MES patients. * means *p* < 0.05, ** means *p* < 0.01. TIME, tumor immune microenvironment; TNBS, triple‐negative breast cancer; TNBC‐IM, TNBC immunomodulatory subtype; TNBC‐MES, TNBC mesenchymal‐like subtype.

In our previous research, TNBCs were classified into four transcriptome‐based subtypes: TNBC‐LAR, TNBC‐IM, TNBC‐BLIS, and TNBC‐MES.^13^ Differences in the components of the TIME among the four subtypes of TNBC were also explored in this study, and details of univariate analysis were shown in Table S3. The PD‐L1^+^ cell density in the IM (0–25 and 0–50 μm) significantly differed between the TNBC‐IM and TNBC‐non IM subtypes (*p* = 0.004 and 0.045), as shown in Figure 5A,B. The level of PD‐L1^+^/CD8^+^ cells in the IM (0–50 and 0–100 μm) was significantly lower in the TNBC‐MES subtype than in the TNBC‐non‐MES subtype (*p* = 0.033 and 0.025), as shown in Figure 5C,D. For further correlation analysis, we assessed the indicators PD‐L1^+^ cells in the IM (0–25 μm) and PD‐L1^+^/CD8^+^ cells in the IM (0–100 μm).

### 3.4 Correlations between the components of the TIME and radiomic features that could reflect subtypes of TNBC

Our previous research developed and validated radiomic models that can fairly distinguish the transcriptome‐based subtypes of TNBC.^15^ The radiomic features SmallAreaEmphasis, LargeDependenceEmphasis, Busyness, Strength, Contrast, SmallDependenceLowGrayLevelEmphasis, LowGrayLevelEmphasis, LowGrayLevelRunEmphasis, MaximumProbability, Minimum, and Kurtosis of Correlation are related to differentiating the IM subtype, while Elongation and Coarseness are helpful for distinguishing the MES subtype.

**FIGURE 6:**
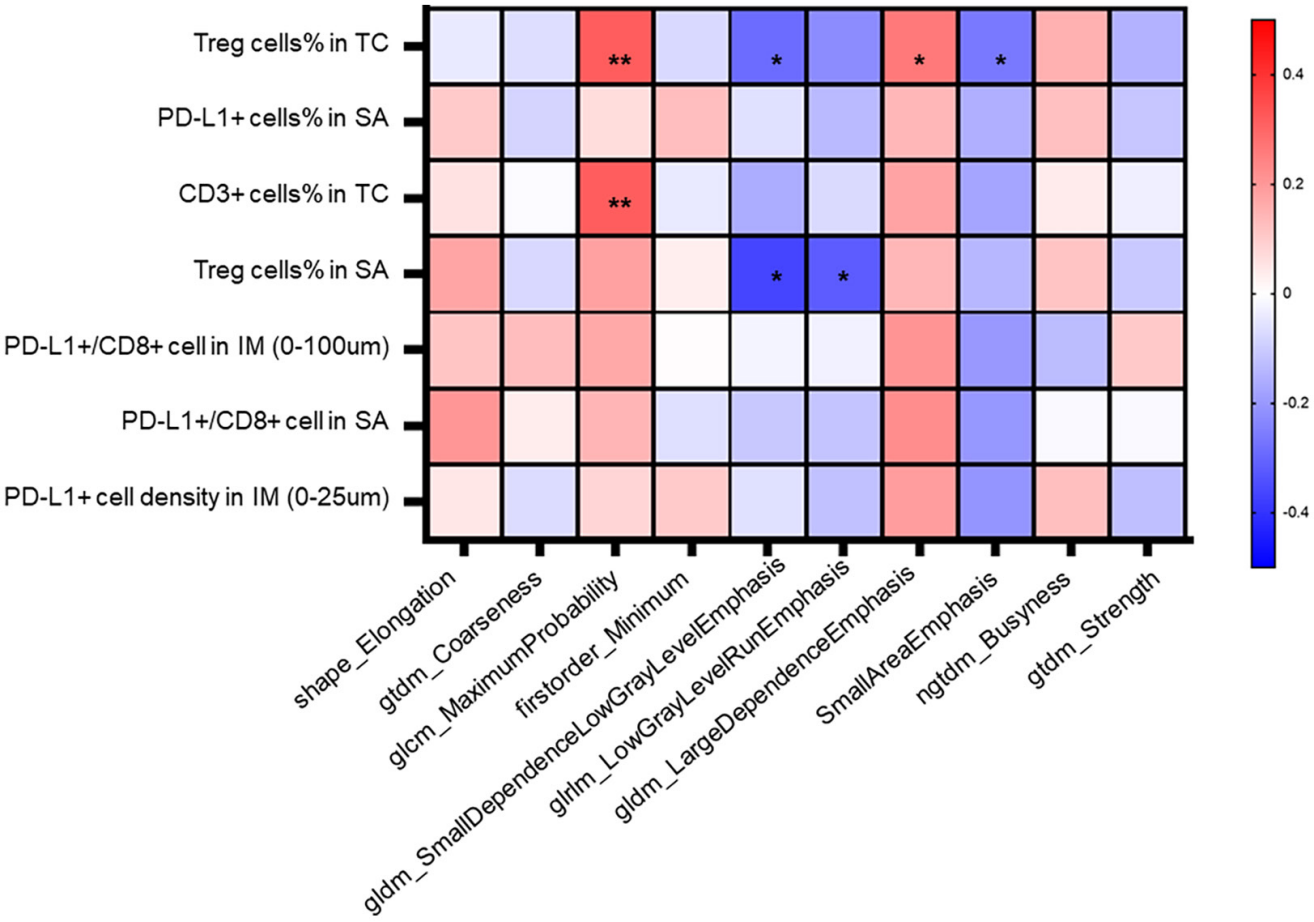
Correlation coefficient matrix between several TIME indicators and MRI radiomic features reflecting TNBC subtypes. * means *p* < 0.05, ** means *p* < 0.01.

In this study, associations between some of the important components of the TIME and the above relevant radiomic features were explored (Figure 6). Glcm_MaximumProbability was positively related to Treg cell % in the TC and CD3^+^ cells in the TC (*p* = 0.007 and 0.008). Ggldm_SmallDependenceLowGrayLevelEmphasis was negatively related to Treg cell % in the TC and Treg cell % in the SA (*p* = 0.048 and 0.012). Gldm_LowGrayLevelRunEmphasis was negatively related to Treg cell % in the SA (*p* = 0.028). SmallAreaEmphasis and ngtdm_Busyness were positively and negatively related to Treg cell % in the TC (*p* = 0.028 and 0.026).

## 4 DISCUSSION

In this research, we demonstrated the correlations between the components of the TIME score and clinical outcome and molecular subtype in TNBC patients. The effective exploration of the TIME in situ at the tissue‐cell level is conducive to the predictive prognosis and subtype classification and personalized treatment of TNBC patients.

In this study, we found a novel correlation between the components of the TIME quantitatively measured by the m‐IHC method and the clinical outcomes in the largest cohort of TNBC patients. The spatial distribution and interactions of Treg, PD‐L1^+^, and CD8^+^ T cells in the TC, SA, or invasive margin (IM) of the TIME were demonstrated to be important prognostic markers for TNBC prognosis. In recent years, increasing attention has been given to changes in the TIME because of the effectiveness of immunomodulatory therapy targeting the PD‐L1/PD‐1 pathway in the treatment of various cancers.^2^, ^3^, ^20^ Moreover, the m‐IHC method and quantitative multiplex immunofluorescence platform have become increasingly useful for the analysis of the spatial distribution and interactions of specific cells in the TIME. Significant positive associations between PD‐L1^+^ cells and CD8^+^ T cells in the TC or infiltration region, evaluated based on the m‐IHC method, and progression‐free survival or response to anti‐PD‐1‐based immunotherapy were demonstrated in metastatic melanoma patients.^21^ In addition, the number of PD‐L1^+^ cells and Treg cells near CD8^+^ T cells identified reduced OS in HPV—oral squamous cell cancer via the m‐IHC method.^19^

In breast cancer, a few studies have also focused on the importance of the spatial distribution and interaction of Treg, PD‐L1^+^ or CD8^+^ T cells in the TIME based on the m‐IHC method. In particular, regarding cell interactions in the TIME, researchers have found that nearly 20% of TNBC patients have PD‐L1^+^ cells on the tumor cell surface and that PD‐L1‐positive tumors have a higher CD8^+^ cell density than PD‐L1‐negative tumors in TNBC specimens.^7^ In the therapy response, infiltrating PD‐1^+^ CD8^+^ cells in the TC and CD8^+^ cells in the SA were found to be associated with pCR after NAC.^11^ Moreover, Hayashi et al. demonstrated that the CD8^+^ PD‐L1^+^ cell number in both the SA and TC was higher in TNBC than in HER2 expression and luminal breast cancer cases. They also found that lower numbers of Treg, CD4^+^ PD‐L1^+^, and CD8^+^ PD‐L1^+^ cells in the SA or TC were associated with lower RFS in 22 TNBC patients.^22^ Similarly, we focused on the important role of Treg, PD‐L1^+^, and CD8^+^ T cells in the TIME for predicting prognosis but in a large TNBC patient cohort, with several novel results in the specific cell subsets, measurement details, and research endpoints. We found a tendency for CD4/PD‐L1 and CD8/PD‐L1 double‐positive cells in the SA or TC to be associated with clinical prognosis but without statistical significance. In addition, we found that lower proportions of PD‐L1^+^ cells in the SA and CD3^+^ cells in the TC were associated with a poor prognosis. Further investigation showed the distribution of the above specific subtypes of immune‐related cells at the infiltrative border and that the normalized PD‐L1^+^/CD8^+^ cell ratio in the IM and SA could predict prognosis. Finally, we established the components of the TIME score, which was calculated from Treg, PD‐L1^+^ and CD8^+^ cells in the TIME evaluated by the m‐IHC method, that were statistically significant in predicting OS, DMFS, and RFS. These findings showed the potential of components of the TIME based on m‐IHC for application in the personalized clinical treatment of TNBC patients.

We particularly found a predictive ability of a higher Treg cell % in both the SA and TC for a positive prognosis in this study of 86 TNBC patients. Traditionally, Treg cells are generally considered negative regulators of antitumor responses.^23^ However, other findings suggest that Treg cells can perhaps be further subdivided into more complex subsets, such as naive‐Treg, effector‐Treg, and non‐Treg cells, by multiplex markers and have complex and different regulatory mechanisms on tumor recurrence and metastasis in colorectal cancers and other cancers.^24^, ^25^ Our findings suggest that it is also necessary to screen suitable markers of different Treg types in TNBC for further study. These findings highlight the need to assess the interspatial distribution of immune and tumor cells for the administration of personalized therapies for TNBC patients.

In this study, we found that the components of the TIME were significantly different in different TNBC subtypes. In our previous studies, TNBC was divided into four subtypes, TNBC‐BLIS, TNBC‐IM, TNBC‐LAR, and TNBC‐MES, according to the transcriptome expression status, and the similarities and differences among the four subtypes of TNBC were investigated from multiplex perspectives.^13^ The TNBC‐IM subtype has elevated immune cell signaling and a high prevalence of TILs in both the SA and TC.^13^ In this research, we found that the PD‐L1^+^ cell density in the IM was higher in the TNBC‐IM subtype than in the other subtypes, which was similar to the finding of a previous study and provided additional rationale for the potential benefit of immune checkpoint blockade treatment. Moreover, we found a significantly lower PD‐L1^+^/CD8^+^ cell ratio in the TNBC‐MES subtype than in the other subtypes. In a previous study, investigators found that TNBC‐MES was the most difficult to identify of the four subtypes, either by specific genomic features or by conventional IHC‐assessed androgen receptor (AR), CD8, FOXC1, and DCLK1 expression.^13^, ^14^ Our findings complement the TNBC subtyping system and might help to promote the exploration of immunotherapy for the TNBC‐MES subtype. In summary, some TIME indicators based on the m‐IHC technique might contribute to the classification of and indicate the choice of precise treatment for TNBC patients, although this needs to be researched in depth.

Our study also innovatively discovered the associations of a few particular indicators of the TIME and MRI radiomic features that could reflect TNBC subtypes. Several other studies have aimed to explore the association between radiomic features and the TIME in breast cancer but have especially focused on TIL evaluation using several other methods. Representing qualitative mammographic image characteristics, Yu et al. found that the six most important radiomic features (uniformity, variance, gray level cooccurrence matrix [GLCM] correlation, GLCM autocorrelation, gray level difference matrix [GLDM] low gray level emphasis, and neighborhood gray‐tone difference matrix [NGTDM] contrast) were significantly different between the low and high TIL groups measured by HE staining in TNBC patients.^26^ On MRI, Fan et al. found that radiogenomic features extracted from tumor and surrounding parenchymal tissues were associated with two clustering samples according to genomic subclone compositions and survival in breast cancer.^27^ Wang et al. found different spatial distributions of TIL measures by sequencing data between high and low radiomic scores based on enhanced MRI in locally advanced breast cancer patients.^28^ In this study, we conducted a novel in situ analysis based on a quantitative multiplex immunofluorescence platform of the relationship between the TIME and radiomic features, focusing on TNBC patients. We demonstrated negative associations between some of the GLDM, gray level run length matrix (GLRLM), and gray level run length matrix (GLSZM) features and positive associations between some of the GLCM and GLDM features and the components of the TIME in TNBC, which could be used to predict clinical prognosis. Our findings might help to explain the biological significance of radiomic features to evaluate the spatial distribution and interactions of particular cells in the TIME from a macro and noninvasive perspective.

A limitation of this study is the lack of a validation cohort due to the particularity of the multiomic information. In the future, our group will expand the sample size to carry out prospective clinical trials to verify these findings. We will also try to promote multicenter research to better validate the stability of these TIME indicators and that radiomic model in TNBC populations. Another limitation is that the associations between components of TIME indicators, TNBC subtypes and radiomic features are preliminary and exploratory. We found only a few different components of the TIME between different TNBC subtypes because of the small sample size. We will further study the relationship between the TIME and TNBC subtype based on more robust statistical methods in large TNBC subtype patient groups. In addition, we will explore more precise indicators of the TIME that could be used to distinguish between different TNBC subtypes and attempt to combine radiomics to assess the TIME of TNBC from a noninvasive and macroscopic perspective.

## 5 CONCLUSION

This study demonstrated that the spatial distribution and interactions of particular cells in the TIME were associated with prognosis in TNBC patients, and we then established the TIME score to predict prognosis. In addition, we found that there were differences between different TNBC subtypes and associations between some components of the TIME and radiomic features reflecting TNBC subtypes. M‐IHC‐based quantitative scoring measures, reflecting TIME spatial location and interaction, could aid in the accurate diagnosis of TNBC in prognosis and classification. These findings could be beneficial for personalized treatment of TNBC patients to obtain better clinical outcomes.

## AUTHOR CONTRIBUTIONS

**Luyi Lin:** Conceptualization (equal); data curation (equal); formal analysis (equal); investigation (equal); writing – original draft (equal). **Haiming Li:** Funding acquisition (equal); methodology (equal); writing – review and editing (equal). **Xin Wang:** Methodology (equal). **Zezhou Wang:** Formal analysis (equal); methodology (equal). **Guanhua Su:** Investigation (equal). **Jiayin Zhou:** Data curation (equal). **Shiyun Sun:** Data curation (equal). **Xiaowen Ma:** Investigation (equal). **Yan Chen:** Project administration (equal); writing – review and editing (equal). **Chao You:** Conceptualization (equal); funding acquisition (equal); project administration (equal); writing – review and editing (equal). **Yajia Gu:** Project administration (equal); resources (equal); supervision (equal).

## FUNDING INFORMATION

This project was supported by the grants from The National Natural Science Foundation of China (82271957, 82271940, 82071878), Natural Science Foundation of Shanghai (22ZR1412500), and Shanghai Anticancer Association SOAR PROJECT (SACA‐AX202109) and Wu Jieping Medical Foundation (320.6750.2021‐06‐37).

## CONFLICT OF INTEREST STATEMENT

The authors have no relevant financial or nonfinancial interests to disclose.

## ETHICS STATEMENT

This study was conducted in accordance with the Declaration of Helsinki (revised in 2013). All analyses were approved by the independent ethics committee/institutional review board of Fudan University Shanghai Cancer Center (050432‐4‐1911D).

## CONSENT TO PARTICIPATE

The written informed consent was obtained from each patient.

## CONSENT FOR PUBLICATION

All authors gave their consent for publication.

## Supporting information


Data S1:

## Data Availability

The data underlying this article will be shared on reasonable request to the corresponding author.
