# Supplementary material for: Components of the tumor immune microenvironment based on m‐IHC correlate with prognosis and subtype of triple‐negative breast cancer
Source: Cancer Med. 2023 Dec 7;12(24):21639–50. doi: 10.1002/cam4.6718 (PMC10757132; doi:10.1002/cam4.6718)
Supplement: Supplementary file 1 — Data S1: [file CAM4-12-21639-s001.docx]

**Methods**

***MRI and radiomic features***

The TNBC patients had undergone breast MR examination before biopsy and treatment. CE-MRI was used to analyze the radiomic features. For MRI, a 1.5-T Aurora, 1.5-T GE or 3.0-T Siemens machine was used. For CE-MRI, 0.1-0.2 mmol/kg Gd-DTPA was injected. For the Aurora, GE, and Siemens CE-MRI series, the repetition times were 5.1 s, 5, 6 s and 5 s, echo times were 1.7 s, 2.9 s, and 3.5 s, slice thicknesses were 3 mm, 1.1 mm, and 3 mm, and flip angles were 15°, 15°, and 10°, respectively.

All images were normalized, and all other phases were coregistered into the first postcontrast phase of CE-MRI. Two types of spatial domain features and sequential features, including shape features, first-order features, textural features, wavelet domain features, enhancement rate features and time-varying curve-based features, were extracted using the PyRadiomics package (voxel size: 0.7, 3, 0.7, 3, and 1.5 mm^3^, ‘binWidth’: 25, version 3.0), 41 implemented in Python (version 3.6) and in-house pipelines. In addition, the least absolute shrinkage and selection operator (LASSO) method was used to select the most useful predictive features from the radiomic cohort (glmnet R package).

In our previous study, we developed and validated radiomic signatures that can fairly differentiate TNBC from other breast cancer subtypes and distinguish molecular subtypes within TNBC (15). MRI radiomic features were selected by the LASSO model via 10-fold cross-validation for identifying TNBC and 9-fold cross-validation for distinguishing different TNBC molecular subtypes in that research. Several important MRI radiomic features reflecting TNBC subtypes, including SmallAreaEmphasis, LargeDependenceEmphasis, Busyness, Strength, Contrast, SmallDependenceLowGrayLevelEmphasis, LowGrayLevelEmphasis, LowGrayLevelRunEmphasis, MaximumProbability, Minimum, Kurtosis of Correlation, Elongation and Coarseness, were further evaluated for their correlations with components of the TIME indicators measured by the m-IHC method, as shown in

**Supplementary Table 1.** Summary of all the index of components of TIME indicators measured and analyzed in this research.

|  | **Percentage of particular cells in TC or SA** |  | CD3+ cells % in TC |
| --- | --- | --- | --- |
|  |  |  | CD4+ cells % in TC |
|  |  |  | PDL1+ cells % in TC |
|  |  |  | Treg cells % in TC |
|  |  |  | CD8+ cells % in TC |
|  |  |  | CD4+ PDL1+ cells % in TC |
|  |  |  | CD8+ PDL1+ cells % in TC |
|  |  |  | CD3+ cells % in SA |
|  |  |  | CD4+ cells % in SA |
|  |  |  | PDL1+ cells % in SA |
|  |  |  | Treg+ cells % in SA |
|  |  |  | CD8+ cells % in SA |
|  |  |  | CD4+ PDL1+ cells % in SA |
|  |  |  | CD8+ PDL1+ cells % in SA |
|  | **Particular cell density in IM** |  | CD4+ cell density in IM (0-25um) |
|  |  |  | CD4+ cell density in IM (0-50um) |
|  |  |  | CD4+ cell density in IM (0-100um) |
|  |  |  | PDL1+ cell density in IM (0-25um) |
|  |  |  | PDL1+ cell density in IM (0-50um) |
|  |  |  | PDL1+ cell density in IM (0-100um) |
|  |  |  | CD8+ cell density in IM (0-25um) |
|  |  |  | CD8+ cell density in IM (0-50um) |
|  |  |  | CD8+ cell density in IM (0-100um) |
|  |  |  | Treg cell density in IM (0-25um) |
|  |  |  | Treg cell density in IM (0-50um) |
|  |  |  | Treg cell density in IM (0-100um) |
|  | **Ratio of particular cells in IM or SA** |  | Treg/CD8+ in IM (0-25um) |
|  |  |  | Treg/CD8+ in IM (0-50um) |
|  |  |  | Treg/CD8+ in IM (0-100um) |
|  |  |  | PDL1+/CD8+ in IM (0-25um) |
|  |  |  | PDL1+/CD8+ in IM (0-50um) |
|  |  |  | PDL1+/CD8+ in IM (0-100um) |
|  |  |  | Treg/CD8+ cell ratio in SA |
|  |  |  | PDL1+/CD8+ cell ratio in SA |
|  | **Normalized particular cells around CD8+ cells in SA** |  | Normalized PDL1+ cells around 0-25μm of CD8+ cells in SA |
|  |  |  | Normalized PDL1+ cells around 0-50μm of CD8+ cells in SA |
|  |  |  | Normalized PDL1+ cells around 0-100μm of CD8+ cells in SA |
|  |  |  | Normalized Treg cells around 0-25μm of CD8+ cells in SA |
|  |  |  | Normalized Treg cells around 0-50μm of CD8+ cells in SA |
|  |  |  | Normalized Treg cells around 0-100μm of CD8+ cells in SA |

**Supplementary Table 2*.*** Several MRI radiomic features reflecting TNBC subtypes demonstrated in our previous study (15) were selected in further correlation analysis with components of TIME indicators in this research.

| **TNBC subtype** | **Radiomic feature** | **Extract ROI** |
| --- | --- | --- |
| IM | SmallAreaEmphasis | Tumor-peritumor |
|  | LargeDependenceEmphasis | Tumor-peritumor |
|  | Busyness | Tumor-peritumor |
|  | Strength | Tumor-peritumor |
|  | Contrast | Tumor-peritumor |
|  | SmallDependenceLowGrayLevelEmphasis | Tumor-peritumor |
|  | LowGrayLevelEmphasis | Tumor-peritumor |
|  | LowGrayLevelRunEmphasis | Tumor-peritumor |
|  | MaximumProbability | Tumor-peritumor |
|  | Minimum | Tumor-peritumor |
|  | kurtosis of Correlatio | Tumor-peritumor |
| MES | Elongation | tumor |
|  | Coarsenes | peirtumor |

**Supplementary Table 3*.*** p value of Univariate analysis of all the components of TIME indicators among TNBC subtypes, including TNBC-IM subtype and NOT, TNBC-MES subtype and NOT, TNBC-BLIS subtype and NOT, and TNBC-LAR subtype and NOT.

|  | **TNBC-IM subtype or NOT** | **TNBC-MES subtype or NOT** | **TNBC-BLIS subtype or NOT** | **TNBC-LAR subtype or NOT** |
| --- | --- | --- | --- | --- |
| CD3+ cells % in TC | 0.320 | 0.822 | 0.086 | 0.594 |
| CD4+ cells % in TC | 0.363 | 0.822 | 0.672 | 0.816 |
| PDL1+ cells % in TC | 0.231 | 0.727 | 0.775 | 0.982 |
| Treg cells % in TC | 0.764 | 0.208 | 0.543 | 0.471 |
| CD8+ cells % in TC | 0.539 | 0.292 | 0.481 | 0.451 |
| CD4+ PDL1+ cells % in TC | 0.231 | 0.871 | 0.645 | 0.885 |
| CD8+ PDL1+ cells % in TC | 0.581 | 0.356 | 0.408 | 0.521 |
| CD3+ cells % in SA | 0.444 | 0.437 | 0.638 | 0.679 |
| CD4+ cells % in SA | 0.920 | 0.881 | 0.985 | 0.982 |
| PDL1+ cells % in SA | 0.375 | 0.990 | 0.625 | 0.625 |
| Treg+ cells % in SA | 0.369 | 0.135 | 0.854 | 0.681 |
| CD8+ cells % in SA | 0.815 | 0.254 | 0.389 | 0.360 |
| CD4+ PDL1+ cells % in SA | 0.549 | 0.832 | 0.941 | 0.850 |
| CD8+ PDL1+ cells % in SA | 0.982 | 0.425 | 0.344 | 0.596 |
| CD4+ cell density in IM (0-25um) | 0.103 | 0.891 | 0.472 | 0.579 |
| CD4+ cell density in IM (0-50um) | 0.176 | 0.911 | 0.543 | 0.665 |
| CD4+ cell density in IM (0-100um) | 0.292 | 0.970 | 0.619 | 0.698 |
| PDL1+ cell density in IM (0-25um) | 0.039* | 0.990 | 0.876 | 0.235 |
| PDL1+ cell density in IM (0-50um) | 0.046* | 0.970 | 0.876 | 0.253 |
| PDL1+ cell density in IM (0-100um) | 0.065 | 0.990 | 0.912 | 0.318 |
| CD8+ cell density in IM (0-25um) | 0.408 | 0.159 | 0.580 | 0.301 |
| CD8+ cell density in IM (0-50um) | 0.363 | 0.176 | 0.600 | 0.271 |
| CD8+ cell density in IM (0-100um) | 0.452 | 0.176 | 0.413 | 0.235 |
| Treg cell density in IM (0-25um) | 0.262 | 0.232 | 0.495 | 0.807 |
| Treg cell density in IM (0-50um) | 0.277 | 0.199 | 0.513 | 0.714 |
| Treg cell density in IM (0-100um) | 0.318 | 0.199 | 0.574 | 0.673 |
| Treg/CD8+ in IM (0-25um) | 1.000 | 0.108 | 0.828 | 0.528 |
| Treg/CD8+ in IM (0-50um) | 0.790 | 0.060 | 0.681 | 0.564 |
| Treg/CD8+ in IM (0-100um) | 0.944 | 0.055 | 0.944 | 0.635 |
| PDL1+/CD8+ in IM (0-25um) | 0.630 | 0.050 | 0.405 | 0.551 |
| PDL1+/CD8+ in IM (0-50um) | 0.840 | 0.033* | 0.540 | 0.636 |
| PDL1+/CD8+ in IM (0-100um) | 0.797 | 0.025* | 0.427 | 0.708 |
| Treg/CD8+ cell ratio in SA | 0.388 | 0.165 | 0.449 | 0.157 |
| PDL1+/CD8+ cell ratio in SA | 0.429 | 0.205 | 0.281 | 0.122 |
| Normalized PDL1+ cells around 0-25μm of CD8+ cells in SA | 0.430 | 0.231 | 0.191 | 0.086 |
| Normalized PDL1+ cells around 0-50μm of CD8+ cells in SA | 0.507 | 0.136 | 0.438 | 0.540 |
| Normalized PDL1+ cells around 0-100μm of CD8+ cells in SA | 0.431 | 0.148 | 0.289 | 0.509 |
| Normalized Treg cells around 0-25μm of CD8+ cells in SA | 0.492 | 0.153 | 0.272 | 0.501 |
| Normalized Treg cells around 0-50μm of CD8+ cells in SA | 0.800 | 0.076 | 0.824 | 0.787 |
| Normalized Treg cells around 0-100μm of CD8+ cells in SA | 0.912 | 0.067 | 0.418 | 0.531 |
